# Gender differential in social and economic predictors of incident major depressive disorder in the Ibadan Study of Ageing

**DOI:** 10.1007/s00127-018-1500-7

**Published:** 2018-02-21

**Authors:** Akin Ojagbemi, Toyin Bello, Oye Gureje

**Affiliations:** World Health Organization (W.H.O) Collaborating Centre for Research and Training in Mental Health, Neuroscience, and Substance Abuse, Department of Psychiatry, College of Medicine, University of Ibadan, P.M.B 5017 (G.P.O), Ibadan, Nigeria

**Keywords:** Gender differences, Unipolar depression, Gender roles, Risk factors, Epidemiological transition, Late-life

## Abstract

**Purpose:**

Working on the hypothesis that the social and economic factors associated with onset of late-life depression operate differently for men and women, we investigated the impact of current social relationships and lifetime occupational attainment on incident major depressive disorder (MDD) assessed in three follow-up waves over a period of 5 years.

**Methods:**

Participants were part of a household multistage probability sample of 2149 Nigerians who were aged 65 years and above. The presence of current and lifetime MDD was assessed using the World Health Organization (WHO) Composite International Diagnostic Interview. Participants’ highest occupational attainment was categorised based on the International Standard Classification of Occupations, while socio-economic positions were estimated using asset-based measures relevant to low-income settings. Current social contacts and participation were assessed using items from the WHO Disability Assessment Schedule.

**Results:**

We found an incidence rate of 120.9 per 1000 persons years (95% CI = 110.4–132.5) among 1394 persons who were free of lifetime MDD and dementia at baseline. Incidence rates were 94.7 (95% CI = 82.5–108.7) and 153.8 (136.3–173.6) per 1000 person years, in men and women respectively. In analyses comparing gender and adjusting for the effect of age, we found that while a lifetime of unskilled occupation (trade: HR = 1.4, 95% CI = 1.0–2.0, and elementary occupations: HR = 1.5, 95% CI = 1.1–2.1) was significantly associated with incident MDD in men (but not in women), living in a rural location (HR = 1.3, 95% CI = 1.0–1.7) and having no regular contact with family (HR = 2.2, 95% CI = 1.0–4.7) at baseline significantly predicted subsequent onset of MDD in women.

**Conclusion:**

There was a gender differential in the association of social and economic factors with incident MDD in this sample. These findings have implications for the design of early prevention strategies for late-life depression in sub-Saharan Africa.

**Electronic supplementary material:**

The online version of this article (10.1007/s00127-018-1500-7) contains supplementary material, which is available to authorized users.

## Introduction

The social and economic factors that are associated with onset of late-life depression have been observed to operate differently for men and women [1], and may be especially important when considering elderly persons living in more disadvantaged settings across the world [2]. In this regard, those living in low and middle income countries (LMICs) may be at increased risk of late-life depression.

In a previous study by our group [3], rates of new onset major depressive disorder (MDD) in community elderly Nigerians were much higher than those reported from many other countries. In that study, indices of poor social network such as residence in a rural location and having few contacts with friends were the identified risk factors for MDD in late-life. Even though it is likely that the social circumstances of elderly persons in most part of sub-Saharan Africa are worsening due to changes in traditional extended family support-systems, the two indices of poor social network identified in our previous study are among a limited number of social and economic risk factors for incident late-life depression so far investigated in the region. For example, the impact of social participation in family and community activities, a factor known to promote healthy ageing [4], is yet unclear. Additionally, the role of lifetime occupational attainment which may be an important proxy for longer term economic status [5] remains unknown. An additional gap in the literature is the investigation of how these contextual factors operate in elderly men and women living in a sub-Saharan African context. In view of recent progress in the development of preventive psychosocial interventions for late-life depression in more developed countries [6], enquiries about the role of these previously unexplored indices of social and economic attainment and their differential effect by gender may help identify targets for tailored psychosocial interventions for elderly persons living in sub-Saharan Africa (SSA).

In the present study, we describe incidence of late life MDD assessed in three follow-up waves over a 5-year period in a geographical area inhabited by about a quarter of the Nigerian population at the time of investigation. Specifically, the relationships of several social and economic risk factors to incident MDD as well as their differential effect in men and women were investigated. Our main hypothesis was that the social and economic factors associated with onset of late-life depression in Nigeria and SSA will operate differently for men and women.

## Methods

The Ibadan Study of Ageing (ISA) is a community-based prospective survey of the health and well-being of elderly persons (aged 65 years and over) from eight neighbouring states in South-West (Ondo, Ekiti, Osun, Lagos, Ogun, and Oyo) and North-Central (Kogi and Kwara) regions of Nigeria: an area inhabited by over 25 million people or about 22% of the Nigerian population at the time of study. The methodology of ISA has been fully described [7]. We provide here a brief overview of the methods relevant to the objectives of the present study.

## Sample selection, recruitment and follow-up

From 15 strata, based on state and urban versus rural location, 43 Local Government Areas were selected as the primary sampling units (PSUs). Secondary sampling units (SSUs), defined by enumeration units of 50–70 housing units, were selected from each PSU (172 SSUs in total). A simple random sample of 17 households with individuals aged 65 years or over was then selected from each SSU. We selected one respondent per household and when more than one elderly person was eligible for study (eligibility criteria were being aged 65 years or older and fluent in the language of the study, Yoruba), the Kish table selection method was used to select the one respondent [8]. Up to five visits were made to contact the selected individual, and there was no replacement for those who could not be contacted or who refused to participate in the study. On the bases of our selection procedures, full baseline assessments were conducted on 2149 respondents between November 3, 2003, and August 27, 2004. Respondents were informed about the study, invited to participate, but also assured of their right to decline. Participants were those who provided consent, mostly verbal (either because of illiteracy or by choice), before interviews were conducted. Non-response was predominantly due to non-availability after repeated visits (14%), interviewers unable to trace the original address (4%), death (3%), and physical incapacitation (2%) and rarely due to refusal (2%). Three annual follow-up waves were implemented in 2007, 2008 and 2009.

The survey was approved by the University of Ibadan/University College Hospital, Ibadan Joint Ethical Review Board.

## Measures

In 2003/2004, interviewer-administered surveys were used to assess a range of domains including sociodemographic details, highest occupation attained in the lifetime, number of household items, social networks and engagement in family/community activities, depression, dementia, chronic conditions, physical disability, and lifestyle risk factors. All instruments used in the study were translated into the local Yoruba language (using the iterative back-translation method) and subjected to cultural adaptation.

### Ascertainment of major depressive disorder (MDD)

Assessments for MDD were carried out at baseline and at each follow-up waves using the World Mental Health Survey version of the World Health Organisation (WHO) Composite International Diagnostic Interview (CIDI) [9] and diagnosed according to DSM-IV criteria [10]. The DSM-IV organic exclusion rules were imposed in making the diagnosis of MDD. Incident cases in each wave of follow-up were the number of subjects meeting criteria for MDD for the first time during the corresponding wave. These were ascertained after censoring cases of MDD occurring in the past 12-months or in the lifetime of the respondents determined in the preceding waves. Cases with dementia, ascertained as described below, were additionally excluded in estimating incident MDD.

#### Diagnosis of dementia

The 10-Word Delay Recall Test (10-WDRT) learning list adapted from the Consortium to Establish a Registry of Alzheimer’s Disease (CERAD) ten-word learning list [11] was used to screen while a follow-up second-stage assessment was conducted with the use of the Clinician Home-based Interview to assess Function (CHIF) [12]. The CHIF is a ten-item semi-structured home interview schedule that evaluates respondents’ higher cognitive function by assessing their knowledge of how to perform instrumental activities of daily living. The ten-item instrument collects information about personal history, cooking/food preparation, shopping, finances, medicines, religious attendance, communication with friends/relatives, social and family roles, organization of home/personal clothing, and recognition/awareness. Each item is scored from 0 to 2, with 2 indicating good performance. With a score range of 0–20, a cut-off point of 17/18 provides the best trade off between sensitivity (89.5%) and specificity (68.5%). The 10-WDRT and CHIF were administered independently by different interviewers, usually within 48 h of one another. A psychiatrist reviewed all available information to determine the presence or absence of dementia. The information included scores on the 10-WDRT (< 2) and CHIF (< 18), interviewer’s observations (of respondent’s memory and language), reported functional status (commonly supplemented by key informant’s report), as well as the temporal relationship of the onset of any co-occurring depressive disorder.

### Measures of economic status

#### Number of household possessions

Direct measurement of economic status was achieved using an inventory of 21 household and personal items. This is a validated method for estimating economic status (wealth) in low-income environments [13]. Participants’ economic status was calculated as the ratio of the total number of possessions they had relative to the median number of possessions of the overall sample, and classified as low (≤ 0.5); low-average (> 0.5–1.0); high-average (> 1.0–2.0) or high (> 2).

#### Living condition

The living condition of households in the baseline survey was ascertained using measures such as the type of material used for the floor and walls of the house, as well as sources of water supply and energy for cooking and lighting. These variables have previously been reported as important proxies of the economic status of the elderly in developing countries [13, 14].

#### Participants’ highest occupational attainment

We asked participants about the highest occupation they attained in their lifetime. Responses were categorised based on the International standard classification of occupations (ISCO-08) [15]. Participants in the ISA were broadly distributed across three major occupational categories: (1) elementary occupations, for example, office messengers, labourers, gardeners and subsistent farmers; (2) trade which was mostly small-scale; and (3) semi-skilled or skilled work such as commercial farming, professional drivers and trained machine operators.

### Social relationship

Social network was assessed with the relevant items in the CIDI [9]. The items enquire about the frequency of respondent’s contact with family members who did not live with the respondent as well as the frequency of contact with friends. The response options provided in the CIDI are 1 (nearly every day), 2 (3–4 days per week), 3 (1–2 days per week), 4 (1–3 days a month), 5 (less than once in a month), 6 (never). In this report, we have dichotomized the responses to contacts that are less than once in a month and those more than once in a month.

Social participation was assessed using items derived from the WHO Disability Assessment Schedule, version 2 (WHO-DAS II) [16]. Participants were asked the following two questions: “During the last 30 days, how much did you join in family activities such as eating together, talking with family members, visiting family members, working together?” and “During the last 30 days, how much did you join in community activities such as festivities, religious activities, talking with community members, working together?” Answers were rated as 1 (not at all), 2 (a little bit), 3 (quite a bit), and 4 (a lot). In this study, participants who answered “not at all” to either question were rated as having poor social participation.

### Other baseline risk factors

Residence was classified as rural (< 12,000 households), semi-urban (12,000–20,000 households) or urban (> 20,000 households).

Participants were asked to rate their overall health on the day of interview as very good, good, fair or bad using the WHO-DAS II [16]. The presence of chronic conditions such as arthritis, stroke, angina, diabetes, chronic lung disease, or cancer was ascertained using standard symptom-based questions [17]. Participants were classified as ever having smoked or not, and ever using alcohol or not based on self report. The short form of the International Physical Activity Questionnaire (IPAQ) [18] was used for the assessment of physical activity. The IPAQ measures physical activity across all domains of leisure-time, work, transportation, and household tasks. A summary indicator was used to categorize the respondents into three levels of physical activity: low (physically inactive), moderate, and high levels of physical activity. These categories were based on standard scoring criteria described in the IPAQ scoring protocol.

Over the course of interview, three measurements of mid-upper arm circumference (MUAC) were also carried out at intervals. Following references developed in a sub-Saharan African older adult population [19], we used measurements of the MUAC to derive appropriate weight categories: normal weight, underweight, overweight, and obese.

### Statistical methods

Socio-demographic characteristics of participants in the incident cohort, who could not be followed up in 2009, were compared with those who were followed up using Chi-square test. We implemented corrections accounting for survey design [20] in conducting these comparisons.

Person years at risk were calculated using the actuarial adjustment approach of a life table. This was determined as the period between baseline and follow-up assessments for those who did not develop MDD during follow-up, and between baseline and the mid-point of each interval for those who developed the disorder in the corresponding wave, or were censored because of being lost to follow-up, or had died by the corresponding wave of follow-up. The sensitivity of our analysis to the actuarial method was investigated using the product limit estimator assumption for Kaplan–Meier analyses. To achieve this, we calculated person years for those who developed MDD or were censored using the interval from baseline to the last wave in which the person was followed up. The incidence rates with 95% confidence interval for both actuarial and Kaplan–Meir approaches are presented.

Specific incidence estimates for each baseline risk factors were calculated by dividing the number of incident cases of MDD by the person years contributed.

We used the entire 2003/2004 cohort who was free of both MDD and dementia in estimating the relationship between baseline characteristics and incident MDD during the follow-up period. The Cox regression model for time invariant explanatory variables was applied to derive estimates of hazard ratios (with 95% CI) assuming proportional hazards. We first performed an unadjusted analysis. Next, we adjusted for the effect of age.

Data were analysed using STATA version 13.0 [21]. The survey commands in Stata were used to account for the study sampling scheme. A significance level of 0.05 was used throughout the analyses. Coefficient estimates and 95% confidence intervals are presented for the regression models.

## Results

**Fig. 1:**
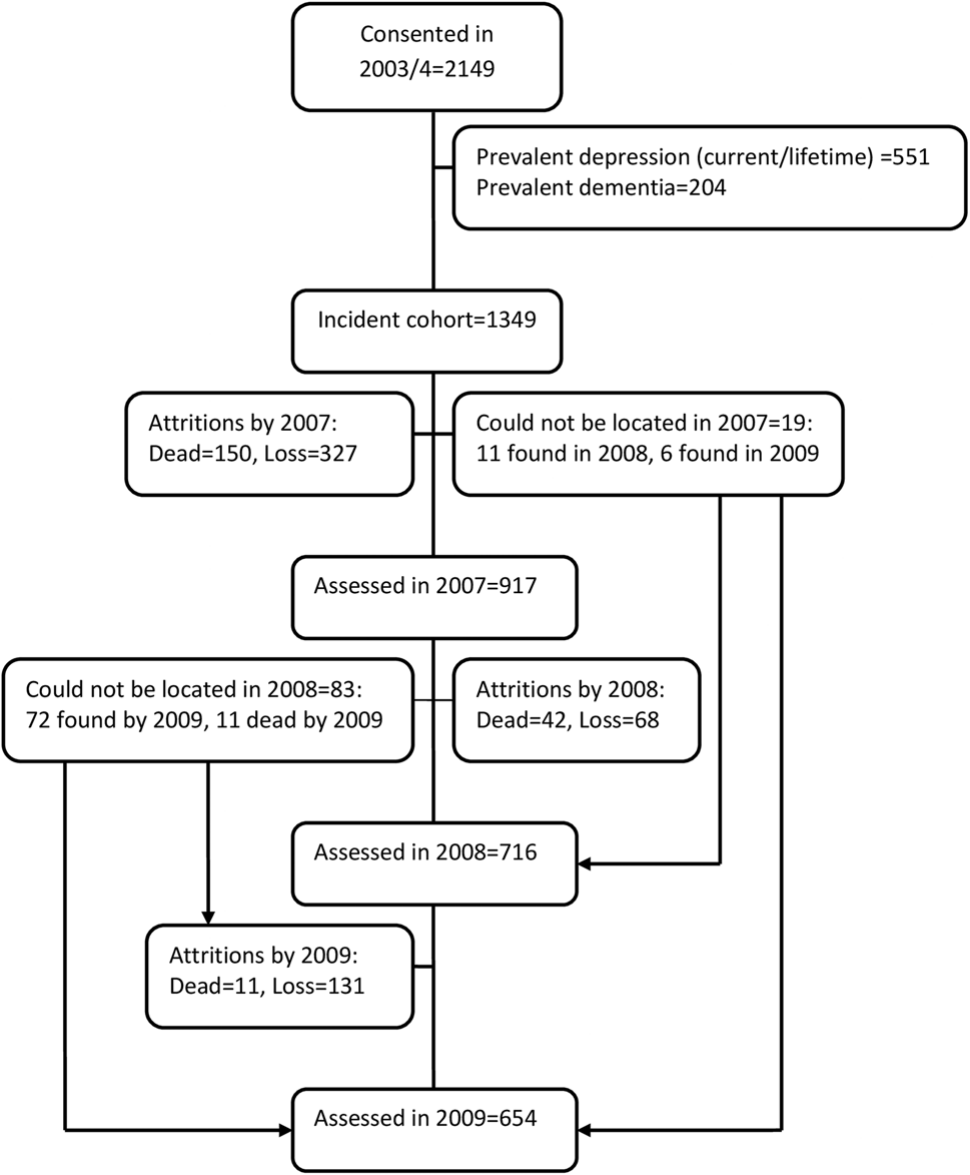
Flow chart for the study

A total of 2149 participants, out of 2873 who were eligible, gave consent and participated in 2003/2004. Of these, 1394 persons were free of both lifetime and current MDD and dementia. They constituted the incident cohort that was subsequently followed up (Fig. 1). Together, they contributed a total of 3837 risk years estimated using the actuarial approach. The corresponding number of risk years using the Kaplan–Meier approach was 4082.

The mean age of the incident cohort was 74.6 (± 9.0) years. Table 1 describes the characteristics of participants who were followed up compared with those who were lost to follow-up or had died before the last follow-up assessment in 2009.

**Table 1:** Baseline characteristics of the incident depression cohort

Characteristics	Incomplete follow-up (*n* = 537)		Completed follow-up (*n* = 857)		Statistics		
	*n*	%	*n*	%	*χ* ^2^	*df*	*P* value
Age (years)							
65–69	197	42.9	271	36.6	2.44	3	0.078
70–74	119	27.3	230	32.0			
75–79	65	13.8	116	18.0			
80+	156	16.1	240	13.4			
Gender							
Male	264	62.1	456	65.0	1.63	1	0.220
Female	273	37.9	401	35.0			
Place of residence							
Urban	140	26.9	207	23.1	1.30	2	0.286
Semi-urban	193	39.2	354	42.3			
Rural	204	33.9	296	34.5			
Formal education (years)							
≥13	47	9.9	70	8.1	0.43	3	0.659
7–12	66	13.0	116	14.0			
1–6	125	22.9	211	25.3			
0	299	54.2	460	52.7			
Economic status							
High	37	8.7	95	15.0	5.15	2	0.017
Average	303	64.1	517	63.2			
Low	197	27.2	245	21.8			
Occupational attainment							
Elementary	211	49.1	324	45.8	0.43	2	0.641
Trade	202	37.2	355	38.4			
Semi-skilled/higher	56	13.8	100	15.8			
Regular social contacts							
Present	504	99.7	833	99.9	3.36	1	0.086
Absent	4	0.3	1	0.1			
Regular social participation							
Present	473	94.9	778	96.2	0.80	1	0.383
Absent	34	5.1	51	3.8			
Self report of health status							
Poor	32	4.9	54	6.2	0.98	1	0.338
Good	478	95.1	781	93.8			
Sub-syndromal depression							
Present	10	2.4	22	2.8	0.24	1	0.633
Absent	527	97.6	835	97.2			
Functional disability							
Absent	493	93.2	761	92.5	0.20	1	0.658
Present	44	6.8	96	7.6			
Medical comorbidities							
Absent	267	54.7	453	57.3	0.42	1	0.528
Present	219	45.3	362	42.7			
Ever smoked							
No	278	55.4	483	62.1	3.94	1	0.064
Yes	220	44.6	321	37.9			
Ever drank							
No	259	51.1	459	52.5	0.15	1	0.708
Yes	222	49.0	358	47.5			
Weight							
Normal weight	254	48.7	412	48.5	0.47	3	0.652
Under weight	47	8.8	78	6.9			
Over weight	135	27.8	214	27.9			
Obese	89	14.7	147	16.7			
Levels of physical activity							
Low	191	29.9	302	28.1	0.18	2	0.822
Moderate	239	48.4	408	50.2			
High	107	21.7	147	21.7			

All percentages are weighted according to study design

Women in the incident cohort were more likely to be older than 80 years and more likely to belong to the lowest economic group (see supplementary Table 1). Social relationships were not significantly differentiated by lifetime occupational-status.

We identified 312, 131, and 21 incident cases of MDD in the 2007, 2008, and 2009 waves, respectively, producing an annual incidence rate of 120.9 per 1000 person years (95% CI = 110.4–132.5) using the actuarial approach. Using the same method of estimation, the annual incidence rates in men and women were 94.7 per 1000 person years (95% CI = 82.5–108.7) and 153.8 per 1000 person years (95% CI = 136.3–173.6), respectively.

We note that the actuarial and Kaplan–Meier approaches produced similar results (Table 2). In analyses across gender categories and adjusting for the effect of age, we found that while a lifetime of unskilled occupation (small-scaled trade: HR = 1.4, 95% CI = 1.0–2.0, and elementary occupation: HR = 1.5, 95% CI = 1.1–2.1) was associated with incident MDD in men (but not in women),living in a rural location (HR = 1.3, 95% CI = 1.0–1.7) and having no regular contact with family at baseline (HR = 2.2, 95% CI = 1.0–4.7) significantly predicted subsequent onset of MDD in women, but not in men (Table 3). None of the other baseline features was associated with incident MDD.

**Table 2:** Kaplan–Meier validation of the actuarial method showing the 5 years incident of major depressive disorder and associations with age and gender in the Ibadan Study of Ageing

Baseline characteristics	Actuarial method			Kaplan–Meier method		
	No of incident cases	Incidence rate per 1000 person-years (95% CI)	Unadjusted HR (95%)	No of incident cases	Incidence rate per 1000 person-years (95% CI)	Unadjusted HR (95%)
Overall	464	120.9 (110.4–132.5)	–	464	113.7 (103.8–124.5)	–
Age (years)						
65–69	156	120.6 (103.1–141.1)	Reference	156	118.4 (101.2–138.5)	Reference
70–74	114	112.9 (94.0–135.7)	1.1 (0.8–1.5)	114	105.1 (87.4–126.2)	1.0 (0.8–1.4)
75–79	69	133.2 (105.2–168.7)	1.2 (0.9–1.7)	69	122.3 (96.6–154.9)	1.1 (0.8–1.6)
80+	125	123.1 (103.3–146.7)	0.9 (0.7–1.2)	125	112.1 (94.1–133.6)	0.9 (0.7–1.2)
Gender						
Male	202	94.7 (82.5–108.7)	Reference	202	91.3 (79.6–104.8)	Reference
Female	262	153.8 (136.3–173.6)	1.6 (1.2–2.0)*	262	140.1 (124.1–158.1)	1.6 (1.3–2.0)*

*< 0.05

**Table 3:** Five years incidence of major depressive disorder and associations with social and economic risk factors among men and women in the Ibadan Study of Ageing

Baseline risk factors	Men				Women			Adjusted^a^ HR (95%)
	No of incident cases	Incidence rate per 1000 person-years (95% CI)	Unadjusted HR (95%)	Adjusted^a^ HR (95%)	No of incident cases	Incidence rate per 1000 person-years (95% CI)	Unadjusted HR (95%)	
Overall	202	94.7 (82.5–108.7)	N/A	N/A	262	153.8 (136.3–173.6)	N/A	N/A
Economic factors								
Education (years)								
13+	14	73.1 (43.3–123.4)	Reference	Reference	22	197.3 (129.9–299.7)	Reference	Reference
7–12	28	91.8 (63.4–133.0)	1.4 (0.6–3.3)	1.4 (0.6–3.3)	33	147.7 (105.0–207.7)	0.6 (0.2–1.6)	0.6 (0.2–1.6)
1–6	48	97.0 (73.1–128.7)	1.5 (0.7–3.3)	1.5 (0.7–3.2)	64	149.0 (116.6–190.4)	0.7 (0.3–1.4)	0.7 (0.3–1.4)
0	112	98.1 (81.5–118.0)	1.7 (0.8–3.8)	1.7 (0.8–3.8)	143	152.4 (129.3–179.5)	0.8 (0.4–1.5)	0.8 (0.4–1.5)
Occupational attainment								
Skilled	35	82.6 (59.3–115.1)	Reference	Reference	8	166.7 (83.3–333.3)	Reference	Reference
Trade	35	90.0 (64.6–125.3)	1.4 (1.0–2.0)	1.4 (1.0–2.0)*	169	156.9 (135.0–182.5)	0.9 (0.3–2.4)	0.9 (0.3–2.3)
Elementary	112	96.9 (80.5–116.6)	1.5 (1.1–2.1)	1.5 (1.1–2.1)*	62	159.6 (124.4–204.7)	1.2 (0.5–2.9)	1.2 (0.5–2.8)
Household assets (number)								
3–10	147	106.3 (90.5–125.0)	Reference	Reference	153	166.1 (141.8–194.6)	Reference	Reference
< 3	33	74.7 (53.1–105.0)	0.8 (0.5–1.3)	0.8 (0.5–1.3)	88	129.5 (105.1–159.6)	0.8 (0.7–1.0)	0.8 (0.7–1.1)
> 10	22	71.2 (46.9–108.1)	0.8 (0.5–1.1)	0.8 (0.5–1.1)	21	204.9 (133.6–314.2)	1.2 (0.8–1.9)	1.3 (0.8–1.9)
Place of residence								
Urban	39	79.1 (57.8–108.3)	Reference	Reference	68	157.8 (124.4–200.1)	Reference	Reference
Semi-urban	94	110.4 (90.2–135.1)	1.4 (0.9–2.2)	1.5 (1.0–2.2)	104	143.9 (118.8–174.4)	1.0 (0.8–1.4)	1.0 (0.8–1.4)
Rural	69	87.5 (69.1–110.7)	1.2 (0.8–2.0)	1.2 (0.8–2.0)	90	163.8 (133.2–201.4)	1.3 (1.0–1.7)	1.3 (1.0–1.7)*
Household floor types								
Hard floor	161	96.5 (82.7–112.6)	Reference	Reference	199	152.7 (132.9–175.4)	Reference	Reference
Earthen floor	33	88.9 (63.2–125.1)	0.9 (0.6–1.3)	0.9 (0.6–1.3)	47	160.7 (120.7–213.9)	1.1 (0.8–1.5)	1.1 (0.8–1.5)
Source of water supply^b^								
Unprotected	140	97.2 (82.4–114.7)	Reference	Reference	182	154.0 (133.2–178.1)	Reference	Reference
Protected	56	90.7 (69.8–117.8)	0.8 (0.6–1.1)	0.8 (0.6–1.1)	64	159.0 (124.5–203.1)	1.0 (0.8–1.4)	1.0 (0.8–1.3)
Source of energy for cooking								
Electricity	11	70.7 (39.2–127.7)	Reference	Reference	22	186.4 (122.8–283.2)	Reference	Reference
Paraffin/kerosine	64	99.5 (77.8–127.1)	1.9 (0.8–4.4)	1.8 (0.8–4.3)	71	131.8 (104.5–166.4)	0.6 (0.4–0.9)	0.6 (0.4–0.9)
Firewood	121	96.2 (80.5–115.0)	1.9 (0.8–4.3)	1.9 (0.8–4.3)	154	167.5 (143.0–196.1)	0.8 (0.5–1.1)	0.8 (0.5–1.1)
Source of light supply								
Electricity	91	100.9 (82.2–124.0)	Reference	Reference	112	154.5 (128.4–185.9)	Reference	Reference
Fossil	105	91.1 (75.3–110.4)	0.8 (0.6–1.1)	0.8 (0.6–1.1)	135	157.8 (133.3–186.8)	1.2 (0.9–1.5)	1.2 (0.9–1.5)
Social factors								
Regular contacts with friends								
Yes	184	93.8 (81.2–108.4)	Reference	Reference	212	150.8 (131.8–172.6)	Reference	Reference
No	11	93.6 (51.8–169.0)	0.9 (0.6–1.5)	0.9 (0.5–1.4)	46	197.4 (147.9–263.6)	1.3 (0.9–1.7)	1.3 (0.9–1.7)
Regular contacts with family								
Yes	193	93.7 (81.4–107.9)	Reference	Reference	254	155.7 (137.7–176.1)	Reference	Reference
No	2	114.3 (28.6–457.0)	2.0 (0.6–6.2)	1.9 (0.6–6.1)	4	533.3 (200.2–1421.0)	2.2 (1.0–4.7)	2.2 (1.0–4.7)*
Participation in family activities								
Yes	174	92.5 (79.7–107.3)	Reference	Reference	227	163.5 (143.5–186.2)	Reference	Reference
No	21	109.9 (71.7–168.6)	1.1 (0.6–2.0)	1.1 (0.6–1.9)	28	117.2 (80.9–169.7)	0.8 (0.6–1.2)	0.8 (0.5–1.2)
Participation in community affairs								
Yes	175	91.3 (78.7–105.9)	Reference	Reference	226	157.9 (138.6–179.9)	Reference	Reference
No	20	125.8 (81.2–195.0)	1.4 (0.8–2.3)	1.4 (0.8–2.2)	32	158.0 (111.8–223.5)	1.0 (0.7–1.5)	1.0 (0.7–1.5)

^a^Adjusted for age

^b^Unprotected sources include rivers, stream and unprotected wells

*p < 0.05

## Discussion

In this study, we identified a total of 464 new cases of MDD over 5 years in a cohort of 1349 elderly persons, corresponding to an annual incidence rate of 120.9 per 1000 person years. We found that while living in a rural location and having no regular social contact with family members was important in predicting subsequent MDD in women, men whose highest lifetime occupational attainment was unskilled were more likely to develop new onset of the disorder in old age.

The results reported here confirm our previous estimates suggesting a high incidence of MDD in community elderly Nigerians [7]. This study also clarifies the important social and economic risk factors for incident MDD in this population and how these factors may differ by gender.

We interpret aspects of our results relating to estimates of the risk of incident MDD provided by the social and economic factors with caution. First, even though our estimates of risk posed by these factors are meaningfully precise, they are also relatively small. Secondly, we note that only a few persons with incident MDD in the present study had no regular social contacts with family. Additionally, fewer women, than men, belonged to the higher occupational categories. Due to these observations, and because we had also stratified the study sample by gender, we included only age as covariate in our adjusted models. These limitations may serve to downplay the importance of the social and economic factors identified in the present study at the level of policy and development of preventive interventions for late life MDD for this population. Nonetheless, the signals provided by of our estimates of risk are important especially in the context of on-going social and economic transitions in Nigeria and other similar LMICs. Currently, many younger members of households in developing societies leave home and migrate to areas with better economic prospects. This scenario has the effect of reducing the social network of elderly persons living in affected communities. Those who are unable to maintain regular contacts with family members after they might have moved out of the community for economic reasons may be especially at risk of MDD.

Our finding suggesting that social contact as a risk factor for MDD operates differently for men and women is similar to previous reports from both high [1] and lower income countries [22]. These studies report that at the community level, indices of social relationship comprising information and communication may be more important in predicting onset of depression in women, but not in men. This has been reasoned to be because of the observations that while men may value more of the physical aspects of their environment, women attach a greater importance to the social components [23]. Traditional gender roles are fairly well pronounced in the Nigerian context [24]. While men are expected to work outside the home, women often take up the important social responsibility of ‘home-making’ and provision of emotional support to family members. In this set-up, it may be reasonable to expect that ongoing and functional social relationships with family members may continue to be of value to women even after some family members may have left home for economic reasons.

The finding in the present study that social participation was not associated with incident MDD is in keeping with an earlier report examining the cross-sectional association of the disorder with role impairment in the ISA [7]. In that study, our group found that social role impairment, measured using the Sheehan Disability Scale [25], was the least affected by current MDD compared with, for example, coping with work related activities or doing home related chores [7]. Even so, there are several possible reasons why poor social participation was not associated with incident MDD in the present observational study. Firstly, the positive effects of social participation on depression and well-being in the literature have often depended on both the frequency and subjective appraisal of the quality of such activities [26, 27]. For example, engaging in leisure and productive activities may provide more benefit for self esteem, thereby protecting against depression [26]. In the present study we asked participants questions about how much they joined in social activities. This would suggest more of a quantitative estimation of participation in these activities than a subjective appraisal of their quality and how this may or may not be associated with new onset of MDD. Secondly, in determining our incident cohort we excluded participants who had current MDD and dementia at baseline. Depression and dementia are conditions that may be characterised by reduced interest and ability to participate productively in many of the family and community activities covered in the social relationship protocol for the present study [16]. In this way, we may have systematically excluded many participants with poor social participation, thus creating a ‘ceiling effect’ on the ‘participation variable’ and blunting out any association this might have had on incident MDD.

The second major finding of this study is the association between having an unskilled or elementary occupation (as the highest work-status attained in the lifetime) and incident MDD. Unskilled or elementary work-status is proxy for lower economic status in this regard [5]. Similar to our finding for social contacts, a gender differential was also found for this association. Contrarily, economic status measured using number of current household possessions or living conditions did not show significant association with incident MDD in this study. These results are in keeping with observations in prospective longitudinal studies of economic status and health in old age suggesting that different measures of economic status tend to show different life-course effects on health [28]. Typically, this difference depends on whether the dimension of economic status in question is more or less relatively time-varying [29]. For example, economic measures such as current income or number of assets may show a greater level of fluctuation over the life-course compared with dimensions such as the highest education or occupation attained in the lifetime. Lifetime highest occupational attainment is a composite index embracing education, longer term income, social status, and potential for asset acquisition over-time. Such multidimensional and relatively stable measure of economic status may be more likely to capture longer term economic effect on incident MDD in old age. The health advantages of longer term and relatively stable measure of economic status, such as lifetime highest occupational attainment, is known to accumulate over the life-course [30, 31]. This accumulation may translate to a significant protective advantage against the onset of MDD by the age of 65 or 70 years and thereafter [28, 32]. On the contrary, and in the same depression context, the study by Kim and Durden [28] found that the advantage of relatively time-varying economic-status indicators was relatively weak and convergent as people approached the age of 65 years, and subsequently becoming unobservable thereafter [28].

The gender differential in the relationship between occupational attainment and MDD found in the present study is in keeping with some reports from communities in Western Europe [23] suggesting that occupational factors may be more relevant to the health and well-being of men, while factors in the home environment may be of greater importance for women. To the best of our knowledge, there are no studies from SSA to which our results could be compared. However, the finding in this study that men with an unskilled occupational attainment in their lifetime were significantly more likely to develop MDD in old age is in keeping with the observed gender roles in Nigeria and across many developing societies [24].

This study has strengths and limitations. The large sample size, covering a wide geographical area inhabited by about 22% of the entire Nigerian population at the time of study should allow for a wider generalization of our findings. We have also used standardized ascertainment procedures in identifying our incident cohort and assessing risk factors. To ensure that the MDD identified in the present study are those occurring for the first time after the age of 65 years, we purposively excluded all patients with diagnoses of current or lifetime MDD and dementia at baseline. However, we note that this procedure may also have led to possible underestimation of the relationship between baseline social and economic risk factors and incident MDD. Person years at risk were calculated using the actuarial adjustment approach of a life table with results validated using the product limit estimator assumption for Kaplan–Meier analyses. Similar to every prospective cohort study, there was significant attrition. We observed that those who dropped-out of the study were more likely to belong to the lower economic categories. However, economic status, measured using number of household possessions, did not show significant association with incident MDD in this study. We acknowledge that this significant negative finding could be due to more drop-out participants belonging in a lower economic position based on their asset-possession. An important area of strength of this study is the use of multiple proxies of economic positions, including lifetime work-status. Even though the specific relationship we observed in the present study was that having an unskilled occupation (which is an important proxy for longer term economic position) predicted incident MDD, it is reasonable to assume that, in a general sense and in this SSA population, low social economic position is a relevant factor for MDD in later life.

In concluding, the finding that certain social and economic risk factors for late-life MDD are more important in the Nigerian context than others and that these factors operate differently in men and women is important for early identification of elderly Nigerians who may require preventive interventions for late-life MDD, as well as targets for such interventions. We suspect that our findings among these Nigerian communities are likely to be applicable to other sub-Saharan African communities in which similar social and economic changes are taking place.

## Electronic supplementary material

Below is the link to the electronic supplementary material.

Supplementary material 1 (DOCX 26 KB)
