# Supplementary material for: Gender differential in social and economic predictors of incident major depressive disorder in the Ibadan Study of Ageing
Source: Soc Psychiatry Psychiatr Epidemiol. 2018 Feb 21;53(4):351–61. doi: 10.1007/s00127-018-1500-7 (PMC5862933; doi:10.1007/s00127-018-1500-7)
Supplement: Supplementary file 1 — Supplementary material 1 (DOCX 26 KB) [file 127_2018_1500_MOESM1_ESM.docx]

**Supplementary Table 1: Baseline demographic characteristics of men and women in the incident cohort**

| **Characteristics** | **Men**  **N=720** | | **Women**  **N=674** | | **Statistics** | | |
| --- | --- | --- | --- | --- | --- | --- | --- |
|  | **n** | **%** | **n** | **%** | **ᵡ^2^** | **df** | **P value** |
| **Age group**  65-69  70-74  75-79  80+ | 261  206  83  170 | 40.6  33.4  14.7  11.3 | 207  143  98  226 | 35.6  25.1  19.7  19.6 | 6.99 | 3 | <0.001 |
| **Site**  Urban  Semi-urban  rural | 174  275  271 | 23.9  39.8  36.4 | 173  272  229 | 25.5  43.9  30.6 | 2.19 | 2 | 0.136 |
| **Years of formal education**  >13  7-12  1-6  0 | 67  93  173  387 | 9.5  13.3  24.7  52.5 | 50  89  163  372 | 7.3  14.3  24.1  54.3 | 0.53 | 3 | 0.655 |
| **Economic status**  High  average  low | 91  464  165 | 15.8  66.7  17.5 | 41  356  277 | 7.6  57.8  34.5 | 27.60 | 2 | <0.001 |
| **Occupation**  Elementary  Trade  Semi-Skilled/higher | 384  135  136 | 59.6  19.6  20.9 | 151  422  20 | 23.7  71.7  4.6 | 59.17 | 2 | <0.001 |
| **Current social contacts**  Present  Absent | 692  3 | 99.9  0.1 | 645  2 | 99.8  0.2 | 0.01 | 1 | 0.9346 |
| **Current social participation**  Present  absent | 662  31 | 97.0  3.0 | 589  54 | 93.4  6.6 | 19.74 | 1 | <0.001 |
| **Self reported health**  Poor  Good | 42  653 | 6.0  94.0 | 44  606 | 5.3  94.7 | 0.12 | 1 | 0.733 |
| **Sub-syndromal depression**  Present  Absent | 19  701 | 3.4  96.7 | 13  661 | 1.4  98.6 | 2.95 |  | 0.105 |
| **Functional disability**  Absent  Present | 654  66 | 93.4  6.6 | 600  74 | 91.6  8.4 | 1.90 | 1 | 0.187 |
| **Medical comorbidities**  Absent  Present | 382  297 | 57.3  42.7 | 338  284 | 54.8  45.2 | 0.82 | 1 | 0.379 |

**All percentages are weighted according to study design**
